# Anatomical and Histological Characterization of the Intestine of *Artibeus lituratus*

**DOI:** 10.1111/ahe.70140

**Published:** 2026-06-05

**Authors:** Mário de Oliveira Magalhães, Jhonatan Henrique Lima da Rocha, Rair de Sousa Verde, Mateus de Oliveira Aquino, Janaira Mattos Amorim, Tamyres Izarelly Barbosa da Silva, Francisco Glauco de Araújo Santos

**Affiliations:** ^1^ Pathology and Wildlife Laboratory Federal University of Acre Acre Brazil; ^2^ Laboratory of Infectious Animal Diseases Federal University of Acre Acre Brazil; ^3^ School of Veterinary Medicine and Animal Science University of São Paulo São Paulo Brazil; ^4^ Catraia Environmental Solutions Acre Brazil

**Keywords:** Chiroptera, fruit bat, histomorphology, intestinal tube

## Abstract

The order Chiroptera is highly diverse and is the second richest among mammals. Approximately 181 species are known in Brazil, with varied feeding habits that make them essential for ecological functions such as seed dispersal and plant regeneration. Despite the importance of their ecosystem services, the anatomical and histological aspects of the digestive system of these animals are still little explored. The aim of this study was to describe the anatomical and histomorphological aspects of the intestines of *A. lituratus* . Six individuals were captured in peri‐urban areas of Rio Branco, Acre. After euthanasia, the intestines were removed, fixed in 10% formaldehyde and processed for macroscopic and histological analysis using Haematoxylin–Eosin staining. Macroscopically, it was not possible to accurately distinguish the small and large intestine portions. Microscopically, all the typical intestinal layers were present, with cells, glands and structures compatible with a frugivorous diet. The findings contribute to the knowledge of the species' digestive morphology and provide subsidies for future investigations into physiological adaptations related to its diet and ecology.

## 1 Introduction

The order Chiroptera, represented by bats, is the second most diverse group of mammals, second only to rodents (Burgin et al. 2018). Currently, approximately 1487 species belonging to this order are recognised (Simmons and Cirranello 2025). The name Chiroptera itself has Greek origins, being composed of the terms ‘cheir’ (hand) and ‘pteron’ (wing), which reflects their striking morphological characteristic: the unique capacity for active flight among mammals (Wund and Myers 2023).

Bats are cosmopolitan animals, being absent only in polar regions and isolated oceanic islands. The Phyllostomidae family is endemic to the Neotropical region and stands out for being one of the most diverse of the order, with around 56 genera and 214 recognised species. Among the various species belonging to this family, *A. lituratus* stands out, a frugivorous bat with a wide geographical distribution, found both in conserved environments and in anthropised areas (Santos et al. 2023).

Considering the great variety, it can be seen that although the small and large intestinal segments are present in most bats, *Desmodus rotundus* is the only species with an absent large intestine. In addition, studies have shown that, due to the tubular and relatively uniform anatomy of the intestine in bats, it is not possible to clearly distinguish its different portions (Paksuz and Paksuz 2015).

The intestinal anatomy of bats can vary according to their feeding habits. In frugivorous species such as *A. lituratus* , the small intestine is proportionally longer (≈20 cm) compared to insectivorous or hematophagous species. This characteristic reflects adaptations to flight and diet, considering their need for greater time and contact area for digestion and absorption of more complex components present in fruit (Eisentraut 1950; Robin 1881).

With regard to the large intestine, a striking feature is its relatively short length, an adaptation that contributes to digestive efficiency, compatible with the flight requirements of fruit bats such as *A. lituratus* . Some authors also affirm the absence of structures such as the caecum and caecal appendix, containing only a larger diameter in the final portion, which represents the rectum (Geronimo et al. 2022; Neuweiler 2000).

With regard to the anatomy and histology of the bat intestine, its macro and micromorphological components are still little explored in the literature. Macroscopic analysis provides information on the anatomical topography of the intestines, species‐specific particularities and characteristics of the organ. In addition, histology makes it possible to detect specialised structures, such as villi, crypts and enteroendocrine cells, which play fundamental roles in digestive and metabolic regulation. In bats, this information is important because it helps to elucidate physiological strategies related to the high energy requirements of flight and the rapid digestion of foods such as fruit and nectar (Geronimo et al. 2022).

Therefore, this study combines macro and microscopic analyses to characterise the intestinal morphology of *A. lituratus* .

## 2 Material and Methods

### 2.1 Ethical Principles

The use of animals in this study was authorised by the Biodiversity Authorization and Information System (SISBIO), under registration no. 83196‐1 and approved by the Animal Use Ethics Committee (CEUA) of the Federal University of Acre (UFAC), under opinion no. 31/2022.

### 2.2 Location, Capture Method and Bat Biometrics

Six specimens of *A. lituratus* were captured in a peri‐urban forest fragment located in the municipality of Rio Branco, state of Acre, Brazil (9°57′18.0″ S, 67°48′22.0″ W). The animals were captured using mist nets set at strategic points over a 6‐h sampling period (Calouro et al. 2010). After capture, the animals were packed in cotton bags and transported to the UFAC Animal Pathology Laboratory for species identification and euthanasia.

The biometric data of each animal was duly recorded on an individual form, including species, sex, weight and size. Bat species were identified according to their morphological and morphometric characteristics, taking into account classic methodological keys (Eisenberg and Redford 1999; Wilson and Reeder 2005).

### 2.3 Euthanasia

The animals were euthanised by deepening the anaesthetic plan with ketamine hydrochloride (Vetanarcol, König Laboratories, Brazil), at a dose of 50 mg/kg, administered intramuscularly in the pectoral muscle. The procedure was then concluded with exsanguination, performed by cardiac puncture (Favoretto et al. 2019).

### 2.4 Sample Collection and Storage

To collect the intestines, the abdominal region was sanitised with 70% alcohol, followed by an incision to open the thoracic and abdominal cavities. The intestines were carefully removed and transferred to vials containing 10% formaldehyde solution for 24 h (Suvarna et al. 2019).

### 2.5 Histological Processing and Histomorphometric Analysis

After fixing the tissues, the intestines were described macroscopically, observing their morphological characteristics and the portions classified according to the topographical anatomy of the chiropteran digestive system.

After inferring the location of each portion according to its topographical characteristics, cleavage was carried out. Starting with the small intestine (duodenum, jejunum and ileum), a segment was obtained just after the stomach (duodenum), at the level of the duodenal loop and the other at an interval of 5 cm from the first (jejunum) and 15 cm from the second (ileum). Next, a segment of the rectum was obtained and, as it is described that bats only have a descending colon, another portion was obtained 1 cm above the rectum. Each fragment was about 3 mm long (Adapted from Silva et al. 2020).

The fragments were subjected to standard histological processing and sectioned at a thickness of 4 μm. For each intestinal segment, six slides were prepared, of which four were stained with Haematoxylin and Eosin (H&E), one with Masson's Trichrome and one with Alcian Blue. H&E‐stained sections were used for general histomorphological tissue analysis; Masson's Trichrome staining was used to better differentiate the layers of the intestinal wall, particularly connective and muscular tissues; and Alcian Blue staining was used to identify goblet cells producing acidic mucins (Santos et al. 2021).

Histomorphometric analysis was performed using ImageJ software (National Institutes of Health, USA). Muscular layer thickness was measured in five random fields per intestinal segment, with three measurements obtained per field and results were expressed in micrometres (μm). Goblet cell quantification was performed on Alcian Blue‐stained sections using five random fields per intestinal segment.

## 3 Results

**FIGURE 1:**
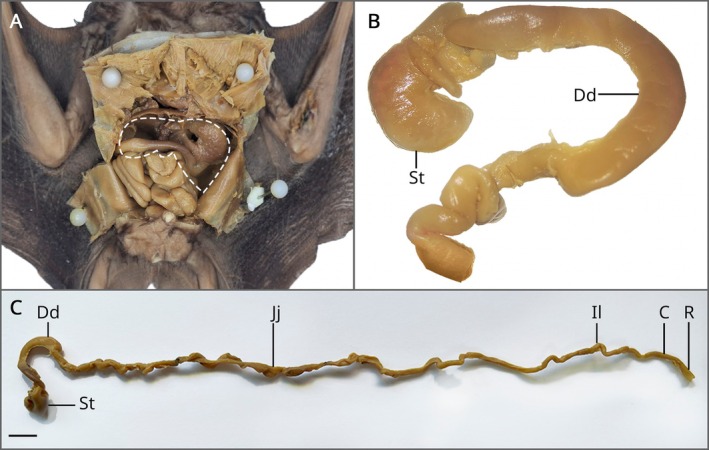
Macroscopic analysis of the gastrointestinal tract of *A. lituratus* . (A) Abdominal cavity with the location of the intestines of *A. lituratus* . The dashed line indicates the liver before removal. (B) Topographical relationship between stomach (St) and duodenum (Dd), highlighting the ‘C’ shaped pancreatic loop. (C) Complete dissection of the intestinal tube, identifying: stomach (St), duodenum (Dd), jejunum (Jj), ileum (Il), colon (C) and rectum (R). Scale bar: 1 cm.

From the specimens evaluated, it was observed macroscopically that it is not possible to clearly distinguish the boundaries of each portion of the intestines, with the exception of the duodenum and rectum, for which a topographical reference was used based on other organs in the abdominal cavity (Figure 1A). For the other portions, the distinction is only possible through histomorphological assessment.

The duodenum is C‐shaped and makes up the first portion of the small intestine (Figure 1B). To infer its topography, we used the stomach as an anatomical reference, which lies just below the diaphragm, on the left side in a ventral aspect, whose pyloric portion is in contiguity with the duodenum, as well as the pancreas.

Located after the duodenal loop, the jejunum is the longest part of the intestine, about 20 cm long and has a tortuous characteristic. It is followed by the ileum, which can only be confirmed histologically as it has no difference from the other parts.

With regard to the large intestine, the rectum was the only macroscopically distinguishable segment because it is the final portion of the digestive tract and is slightly dilated. In addition, there were no structures compatible with the caecum or caecal appendix (Figure 1C).

**FIGURE 2:**
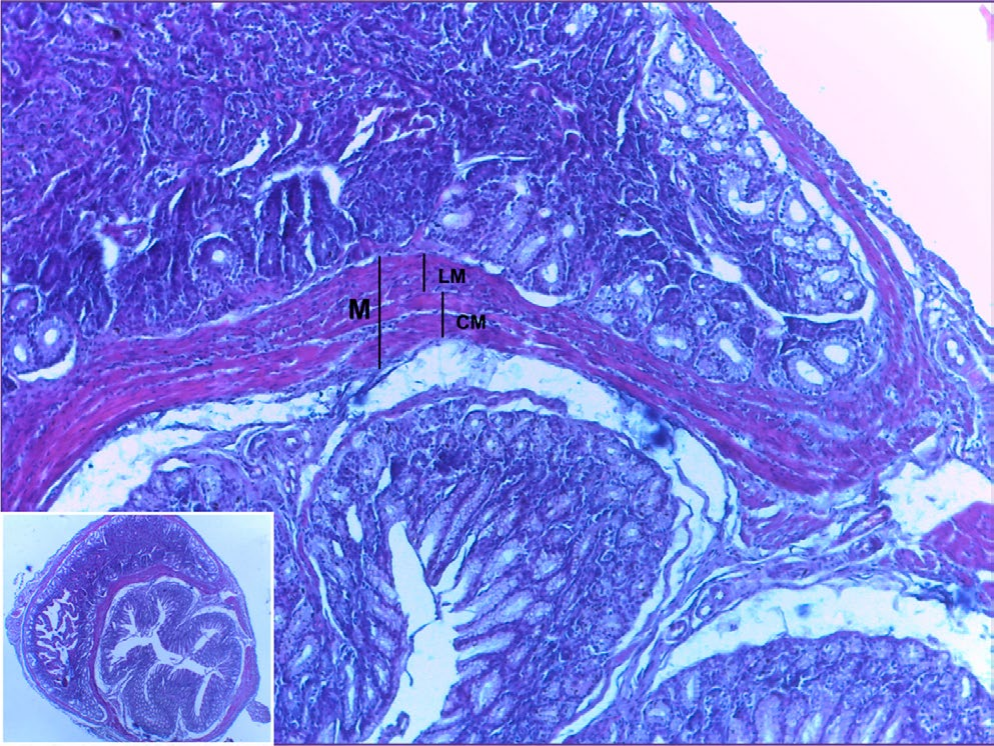
Photomicrograph of the pyloric sphincter region. Divided by smooth muscle (M), the mucosa of the stomach can be seen above and the duodenum below. The musculature is further divided into a longitudinal muscle layer (LM) and a circular muscle layer (CM). HE. 10× and 40× magnification.

Microscopically, in the border region between the stomach and the duodenum, it is possible to find a smooth muscle responsible for regulating the control of intake that passes from the stomach to the intestine, called the pyloric sphincter. The musculature is made up of a longitudinal muscle layer and a circular muscle layer. This sphincter controls the rapid release of this food into the small intestine (Figure 2) (König and Liebich 2021).

The duodenum showed a mucous layer composed of digitiform projections towards the lumen of the organ, increasing the absorption area and acting in conjunction with the microvilli present on the apical surface of the enterocytes. The mucosa was composed of a lining and glandular epithelium, a lamina propria and the muscularis mucosae.

The submucosal layer was thin and formed mainly of loose connective tissue, with the presence of blood vessels, lymphatic vessels, submucosal plexus (Meissner's plexus) and Brunner's glands.

The muscular layer was made up of two smooth muscle portions: one internal, circular, made up of smooth muscle fibres arranged around the lumen and the other external, longitudinal, whose fibres were oriented parallel to the axis of the organ. Connective tissue was observed between these layers, with the presence of the myenteric nerve plexus (Auerbach's plexus).

**FIGURE 3:**
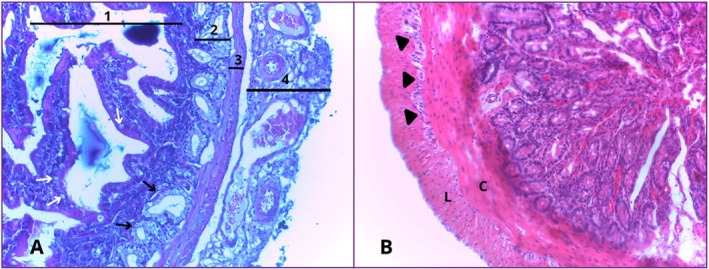
Photomicrograph of the duodenum. (A) The mucosal (1), submucosal (2), muscular (3) and serosal (4) layers can be seen, as well as goblet cells (white arrows) and Brunner's glands (black arrows). (B) The myenteric nerve plexus (arrowhead) can be seen, which lies between the inner circular muscular layer (C) and the outer longitudinal muscular layer (L). HE. 40× magnification.

Externally, the duodenum is lined by a serous layer, consisting of a thin layer of loose connective tissue covered by mesothelium (simple sidewalk epithelium). Blood vessels, lymphatic vessels and collagen fibres were also identified in this region (Figure 3).

The jejunum was the second portion of the small intestine analysed. Microscopically, it has a mucous layer made up of digitiform evaginations that project towards a wider lumen. The lining epithelium is simple, predominantly made up of absorptive epithelial cells with well‐developed microvilli. There is also a lamina propria, made up of loose connective tissue rich in vessels and the muscularis mucosae, made up of smooth muscle fibres.

The submucosal layer is made up of loose connective tissue, which supports the mucosa, as well as blood vessels.

The muscular layer shows smooth muscle organised into two sub‐layers: an inner circular layer and an outer longitudinal layer. Between these sub‐layers, blood vessels, lymphatic vessels and the presence of Auerbach's plexus were identified in various portions. In some regions, only the inner circular layer was more evident.

**FIGURE 4:**
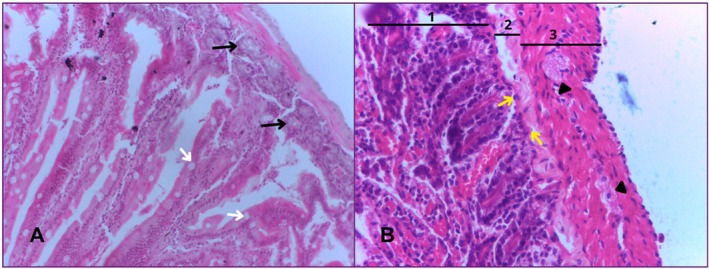
Photomicrograph of the jejunum. In (A) we can see the goblet cells (white arrows) and Brunner's glands (black arrows). Image (B) shows the mucosal (1), submucosal (2) and muscular (3) layers. The myenteric nerve plexus (arrowhead) and the muscularis mucosae (yellow arrows) are also visible. HE. 40× magnification.

The serous layer is thin and made up of loose connective tissue, blood and lymphatic vessels, as well as being covered by a serous membrane made up of simple sidewalk epithelium (Figure 4).

The ileum is the final portion of the small intestine, located anterior to the colon. In the mucosal layer, there is a smaller number of villi, whose shapes vary from digitiform to pyramidal. The lining cells are made up of enterocytes, between which are interspersed goblet cells. The lamina propria is composed of loose connective tissue and delimits the transition between the mucosa and submucosa. The muscularis mucosa is still present, consisting of a thin layer of circularly oriented smooth muscle fibres. The mucosa is reduced compared to the anterior portions of the small intestine, which contributes to a wider lumen. Shorter villi and an increased number of goblet cells compared to the duodenum and jejunum are striking features of this region.

The submucosal layer is made up of loose connective tissue, which supports the mucosa and contains lymphatic vessels and blood vessels.

**FIGURE 5:**
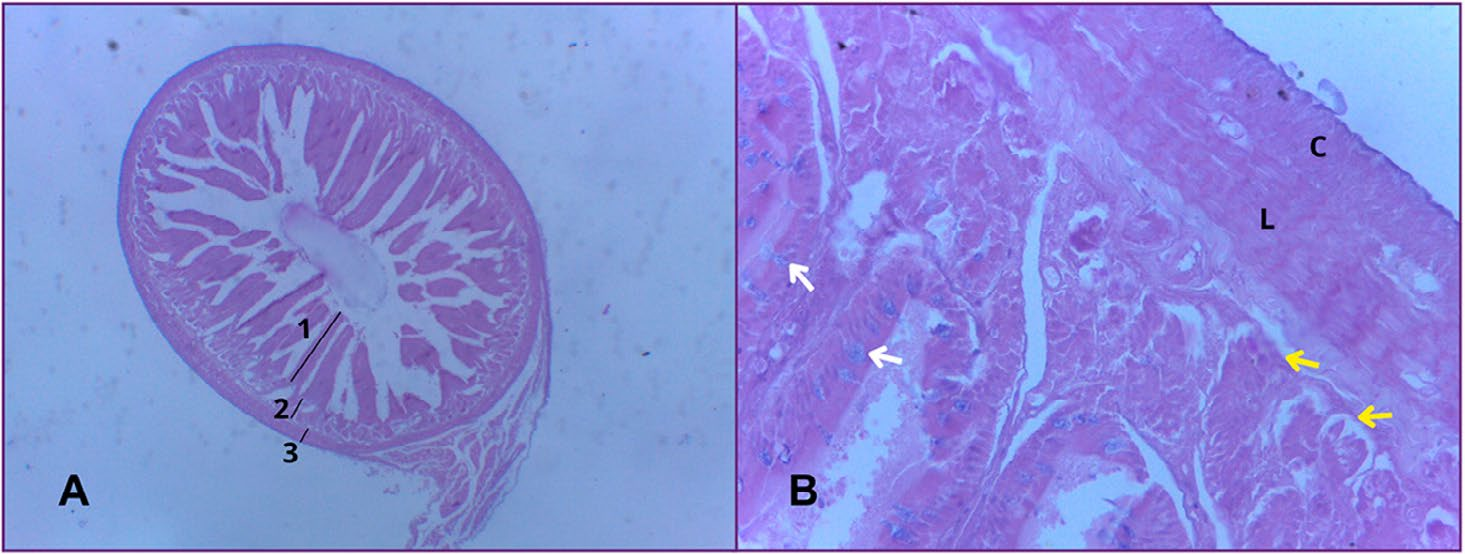
Photomicrograph of the ileum. In image (A) the layers are divided into mucosa (1), submucosa (2) and muscle (3). Image (B) shows goblet cells (white arrows), mucosal muscle (yellow arrows), longitudinal muscle (L) and circular muscle (C). HE. 10× and 40× magnification.

The muscular layer consists of two layers of smooth muscle: an inner circular layer and an outer longitudinal layer, generally of similar thickness along the tube. Between these layers, ganglion cells of Auerbach's plexus were observed. This region is the organ's intrinsic nerve supply. The serosa maintains its typical mesothelium structure over loose connective tissue (Figure 5).

The colon is the first portion of the large intestine to be analysed. In the mucosal layer, villi are absent in some places, making it smooth. It consists of a simple columnar epithelium and a large number of goblet cells, a smaller lamina propria and a muscularis mucosae. Lymphoid follicles and Lieberkühn's crypts are also present.

In the submucosal layer there is connective tissue to support the mucosa, blood vessels, lymphatic vessels and nerve ganglion cells.

In the muscular layer there are two muscle sub‐layers, one inner circular and the other outer longitudinal, which tend to vary in thickness. Auerbach's plexus can also be seen between the muscle layers (inner circular and outer longitudinal).

**FIGURE 6:**
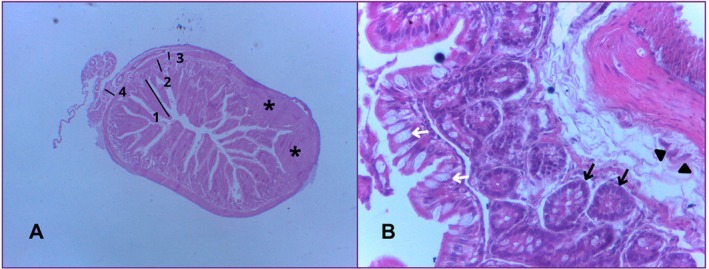
Photomicrograph of the colon, showing the mucosal (1), submucosal (2), muscular (3) and serosal (4) layers, as well as the lymphoid follicles (asterisks) in image (A). In image (B) we can see goblet cells (white arrows), Lieberkühn's crypts (black arrows) and Auerbach's plexus (arrowheads). HE. 10× and 40× magnification.

The serous layer has a simple sidewalk epithelium and underlying it is a layer of very thin loose connective tissue (Figure 6).

The rectum is the final portion of the large intestine; its mucous layer is smooth and absent of villi. It is lined by a simple columnar epithelium and a large number of goblet cells. Presence of lymphoid follicles.

The submucosal layer is made up of loose connective tissue, blood vessels, lymphatic vessels, nerve ganglion cells and lymphatic follicles. There are lymph nodes below the mucosa with a germinal center.

On the muscular side, it has two layers of well‐developed smooth muscle (inner circular and outer longitudinal). Auerbach's plexus can also be seen between the muscle layers.

**FIGURE 7:**
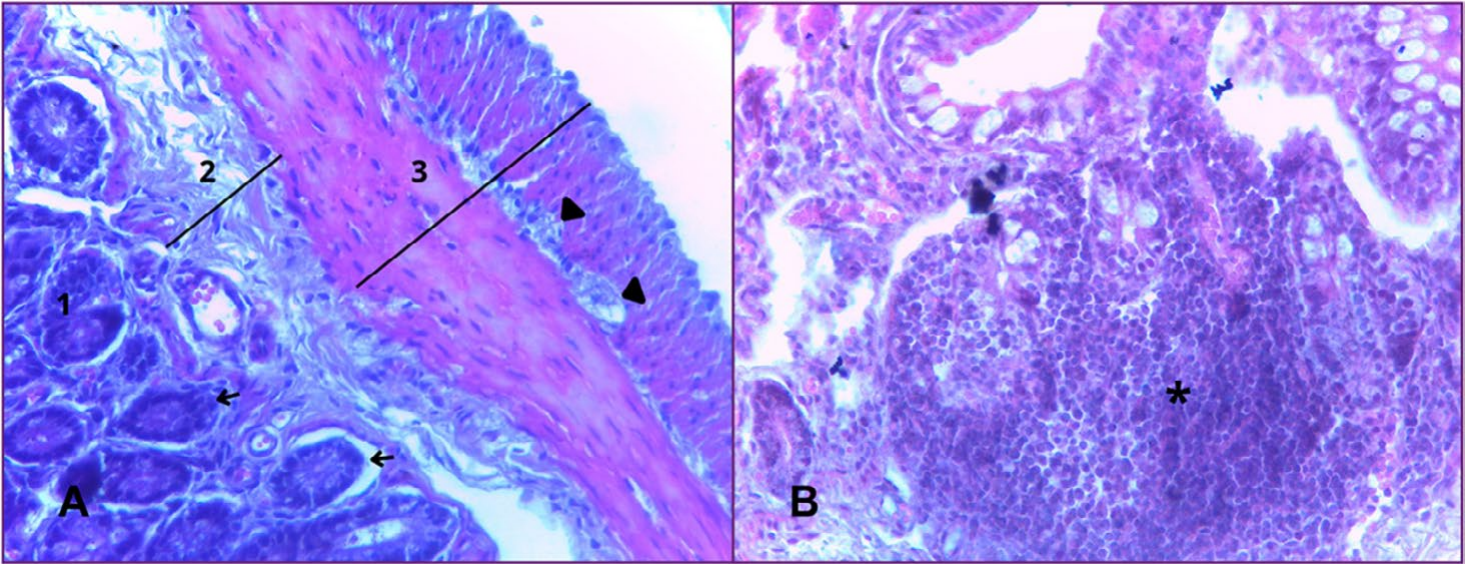
Photomicrograph of the rectum. In this portion, the mucous (1), submucous (2), and muscular (3) layers, Auerbach's plexus (arrowhead) and Lieberkühn's crypts (arrows) can be seen in image (A). Image (B) shows nodular lymphoid tissue (asterisk). HE. 40× magnification.

In the serous layer, there is a simple sidewalk epithelium and underlying it is a layer of loose connective tissue (Figure 7).

**FIGURE 8:**
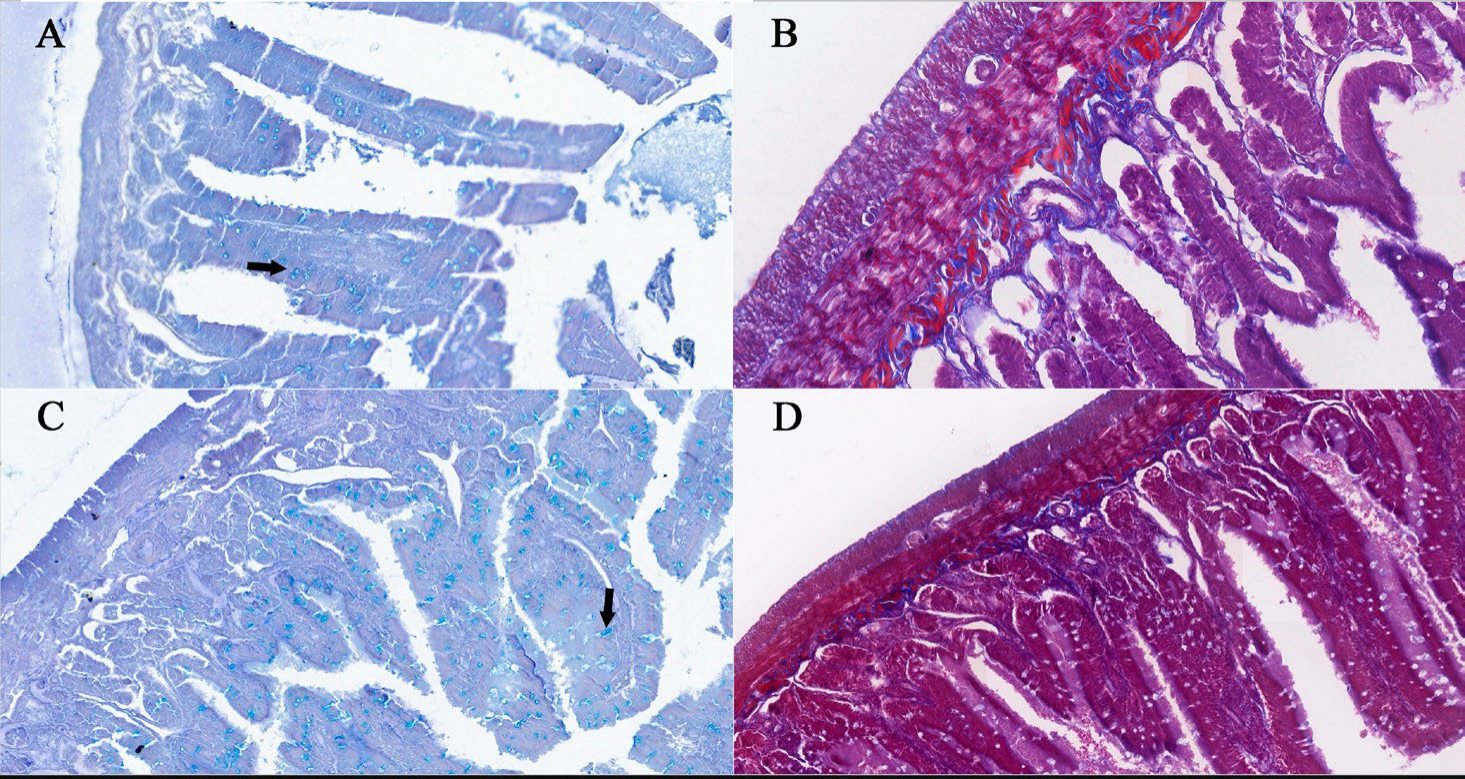
Photomicrographs of the jejunum (A and B) and colon (C and D). In the first column (A and C), Alcian Blue staining highlights goblet cells containing acidic mucins, identified by intense blue staining (arrows). In the second column (B and D), Masson's Trichrome staining demonstrates the organisation of the intestinal wall layers, highlighting submucosal collagen in blue and muscle fibres in red, allowing identification of the muscularis mucosae, inner circular muscle layer and outer longitudinal muscle layer. 40× magnification.

To better illustrate the histological organisation of the intestinal wall and the distribution of goblet cells, representative stained with Alcian Blue and Masson's Trichrome are shown in Figure 8.

The histological findings observed in the intestinal segments demonstrated variations in structural organisation and distribution of specific components along the gastrointestinal tract. Differences were identified regarding the presence and development of intestinal villi, intestinal glands, lymphoid structures and components of the enteric nervous system. A compilation of the main histological characteristics identified in each intestinal segment is presented in Table 1.

**TABLE 1:** Histological characteristics of intestinal segments in *A. lituratus* .

Histological structure	Duodenum	Jejunum	Ileum	Colon	Rectum
Villi	Present	Present	Reduced	Reduced	Absent
Brunner's glands	Present	Present	Absent	Absent	Absent
Meissner's plexus	Present	Present	Present	Present	Present
Lymphoid follicles	Not observed	Not observed	Reduced	Present	Present
Auerbach's plexus	Present	Present	Present	Present	Present
Crypts of lieberkühn	Reduced	Reduced	Reduced	Present	Present

Histomorphometric analysis demonstrated variations in muscular layer thickness along the intestinal tract. The rectum exhibited the greatest muscular thickness (151.40 ± 29.25 μm), whereas lower values were observed in the duodenum (45.44 ± 10.09 μm), jejunum (64.17 ± 8.24 μm), ileum (51.71 ± 10.61 μm) and colon (60.94 ± 15.91 μm), supporting increased muscular development in distal portions of the intestine.

Goblet cell density also varied among intestinal segments. Lower values were observed in the duodenum (19.78 ± 4.24 cells/mm) and jejunum (20.89 ± 5.86 cells/mm), while higher values were recorded in the ileum (45.35 ± 8.59 cells/mm), colon (50.22 ± 22.27 cells/mm) and rectum (46.41 ± 4.34 cells/mm). Overall, goblet cell density increased in distal intestinal regions (Table 2).

**TABLE 2:** Histomorphometric measurements of muscular layer thickness and goblet cell density in different intestinal segments of *A. lituratus* .

Intestinal segment	Muscular layer thickness (μm)	Goblet cells/mm epithelium
Duodenum	45.44 ± 10.09^a^	19.78 ± 4.24^a^
Jejunum	64.17 ± 8.24	20.89 ± 5.86
Ileum	51.71 ± 10.61	45.35 ± 8.59
Colon	60.94 ± 15.91	50.22 ± 22.27
Rectum	151.40 ± 29.25	46.41 ± 4.34

^a^ Values are expressed as mean ± standard deviation (μm).

## 4 Discussion

The present study demonstrates that the intestine of *A. lituratus* exhibits morphological and histological adaptations typical of frugivorous bats, while also presenting particular features that may reflect its ecological niche. Macroscopically, we observed that the topographical anatomy of the intestines of *A. lituratus* is similar to that of other chiropteran species studied, corroborating the findings that, through external morphology alone, there is no clear distinction between the small and large intestines (Silva et al. 2020). The absence of distinctive anatomical landmarks between the intestinal portions, also observed in studies with *A. planirostris* (Geronimo et al. 2022), suggests that anatomical characterisation in fruit bats depends primarily on histological criteria and topographical relationships with other organs.

The transition between the intestines is continuous, without the notable presence of a dilation or structure that delimits them, a characteristic also observed in the bat *Molussus rufus* (Geronimo et al. 2022). Other studies have identified some anatomical differences, such as the V‐shaped stomach described by Yani and Yuliyantika (2019) and the presence of a small dilation separating the intestines, associated with an abundant concentration of lymphoid tissue (Peyer's patches). This last feature is more common in fruit bats and is probably related to their diet (Gadelha‐Alves et al. 2008).

Neither the cecum or the appendix was observed, as described by Geronimo et al. (2022), Zhang et al. (2014) and Paksuz and Paksuz (2015). This finding may be explained by the high energetic demands associated with flight, which may have favoured evolutionary reductions of non‐essential gastrointestinal structures, such as the cecum and appendix. The lack of these structures is reported in most chiroptera, but in a study carried out by Aylward et al. (2019) with an insectivorous bat *Rhinopoma hardwickii* they observed a small caecum in the distal portion of the intestine, which demonstrates that some species can still present a vestigial structure. This is in contrast to fermentative mammals such as horses and rabbits, which have a well‐developed cecum and colon for the digestion of fibres and where there is also a large amount of bacteria and protozoa (Brandi and Furtado 2009). In non‐ fermentative animals, the caecum tends to be simpler and in some even absent, as is the case with *A. lituratus* .

The histological findings of this study reveal that the intestinal architecture of *A. lituratus* follows a general pattern observed in chiropterans (Geronimo et al. 2022; Silva et al. 2020), but with adaptations related to its frugivorous feeding habits and flight requirements. The quadrilaminar pattern (mucosa, submucosa, muscularis and serosa) observed in all portions studied suggests a conserved structural plan within the order Chiroptera.

Histomorphometric analysis demonstrated variations in muscular layer thickness along the intestinal tract, with the rectum presenting the greatest muscular thickness (151.40 ± 29.25 μm), whereas lower values were observed in the duodenum (45.44 ± 10.09 μm), jejunum (64.17 ± 8.24 μm), ileum (51.71 ± 10.61 μm) and colon (60.94 ± 15.91 μm). This greater muscular development in the rectum is likely associated with increased mechanical requirements in distal intestinal portions, contributing to propulsion and elimination of increasingly compact luminal contents. Such structural specialisation may represent an adaptive characteristic related to the regulation of intestinal motility and faecal transport.

Likewise, goblet cell density increased in distal intestinal regions, ranging from 19.78 ± 4.24 cells/mm in the duodenum and 20.89 ± 5.86 cells/mm in the jejunum to higher values in the ileum (45.35 ± 8.59 cells/mm), colon (50.22 ± 22.27 cells/mm) and rectum (46.41 ± 4.34 cells/mm). This progressive increase may reflect a greater requirement for mucus secretion in distal portions of the intestine, where lubrication and epithelial protection become increasingly important. Mucus plays a fundamental role in facilitating the passage of intestinal contents and protecting the epithelium against mechanical stress and microbial interactions. In addition, the reduction of villi and evaginations in the colon and rectum, culminating in a smoother mucosal surface, indicates a lower absorptive demand in these regions and a greater requirement for protection against friction generated by faecal transit (Aylward et al. 2019).

This is the first study to morphologically characterise the small and large intestines of *A. lituratus* . The histological and anatomical simplicity of the *A. lituratus* gut, compared to non‐flying mammals, suggests that the energetic demands of flight favour the reduction of non‐essential structures and optimisation of rapid nutrient absorption. This study lays the foundations for future research into the histology and morphology of bats, addressing issues that have so far been little explored in the intestinal anatomy of these animals. The data generated in this study provide a foundation for future investigations integrating morphology, physiology and ecology, contributing to a broader understanding of the functional diversity of chiropterans and their ecological adaptations.

## Data Availability

Research data are not shared.
